# Supplementary material for: Weather-responsive adaptive shading through biobased and bioinspired hygromorphic 4D-printing
Source: Nat Commun. 2024 Nov 28;15:10366. doi: 10.1038/s41467-024-54808-8 (PMC11604995; doi:10.1038/s41467-024-54808-8)
Supplement: Supplementary file 1 — Supplementary Information [file 41467_2024_54808_MOESM1_ESM.pdf]

Supplementary Information

Weather-Responsive Adaptive Shading through Biobased and Bioinspired Hygromorphic 4D-Printing

Tiffany Cheng, Yasaman Tahouni, Ekin Sila Sahin, Kim Ulrich, Silvia Lajewski, Christian Bonten, Dylan Wood, Jürgen Rühe, Thomas Speck and Achim Menges

Corresponding author: tiffany.cheng@icd.uni-stuttgart.de

Supplementary Table 1: Printing process parameters for the actuating material layer, printed with the biocomposite filament, and restricting material layer, printed with the ASA filament.

Printing process parameter	Actuating material	Restricting material
Feedrate ($\mathrm{mm\,min^{-1}}$)	1600	1200
Flowrate ($\mathrm{mm_{filament}\,mm_{travel}^{-1}}$)	0.105	0.050
Material path offset (mm)	1.050	3.000
Layer offset (mm)	0.200	
Hot-end temperature ($^\circ\mathrm{C}$)	240	
Bed temperature ($^\circ\mathrm{C}$)	70	

Supplementary Table 2: Mechanical characterization of the biocomposite filament used for the actuating material layer. The values represent the mean and standard deviation of measurements from 7 samples per experiment.

Specimen condition	Elastic modulus (MPa)	Elongation at break (%)
Dry (40% RH)	1295.36 ± 177.12	2.9 ± 0.5
Humid (90% RH)	1117.71 ± 97.29	3.0 ± 0.4
Water-immersed	593.25 ± 68.47	5.9 ± 0.5
UV-exposed	1228.62 ± 196.72	1.7 ± 0.2

Supplementary Table 3: Printing time, material consumption, and production cost of the self-shaping shading elements in the building facade.

	**For all windows**	**Per square meter**
**Time and materials consumed**		
Total 4D-printing time (h)	680[a]	18.25[d]
Biocomposite filament (kg)	5.5	0.59
ASA filament (kg)	0.7	0.07
**Production costs**		
Cellulose powder (€)	60	2[e]
PK pellets (€)	700	26[e]
Compounding and extrusion (€)	6400[b]	235[e]
ASA filament (€)	35	4
3D-printer rental (€)	360[c]	40
Sum (€)	**7555**	**307**

[a] Due to four printers working in parallel, the actual 4D-printing time was 170 h (across 17 d).
[b] Four days of lab equipment were booked at a rate of € 1600 per day to produce 16 kg of filament.
[c] Four printers were rented for one month at a rate of € 90 per month per printer.
[d] Adjusted based on the four printers used.
[e] Adjusted according to the amount of material consumed.

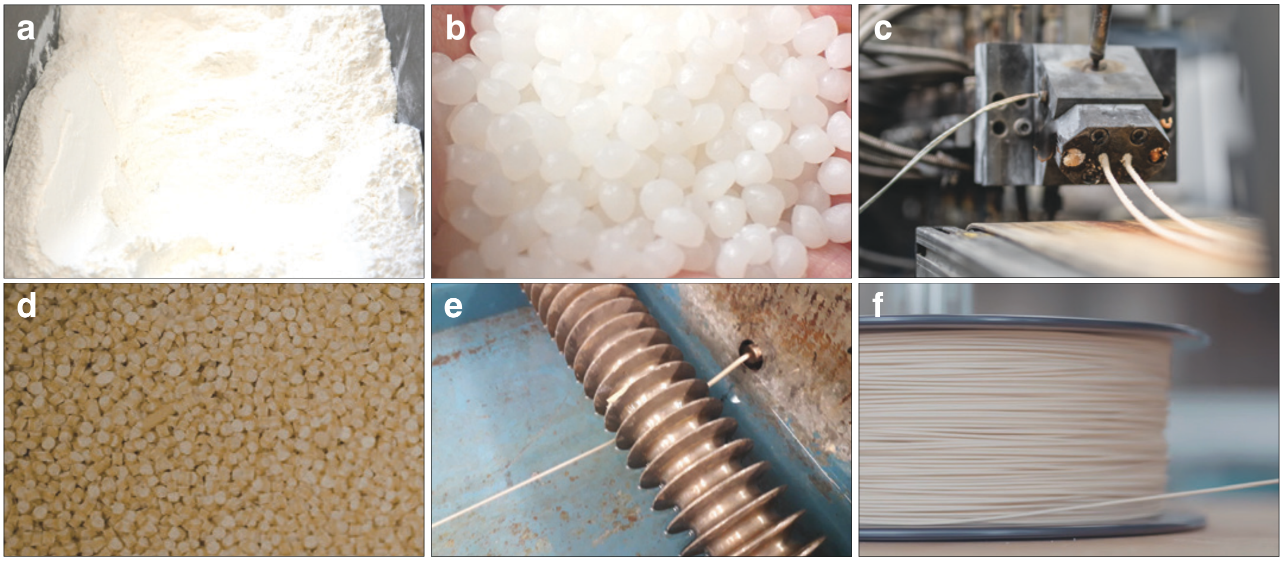

Supplementary Figure 1: Hygromorphic filament production process. (**a**) Cellulose powder and (**b**) polyketone (PK) were (**c**) compounded using a twin-screw extruder. (**d**) The compounded mixture was chopped to form granules, (**e**) and then extruded into filaments. (**f**) The biocomposite filaments were wound into $5\,\mathrm{kg}$ rolls in preparation for 4D-printing.

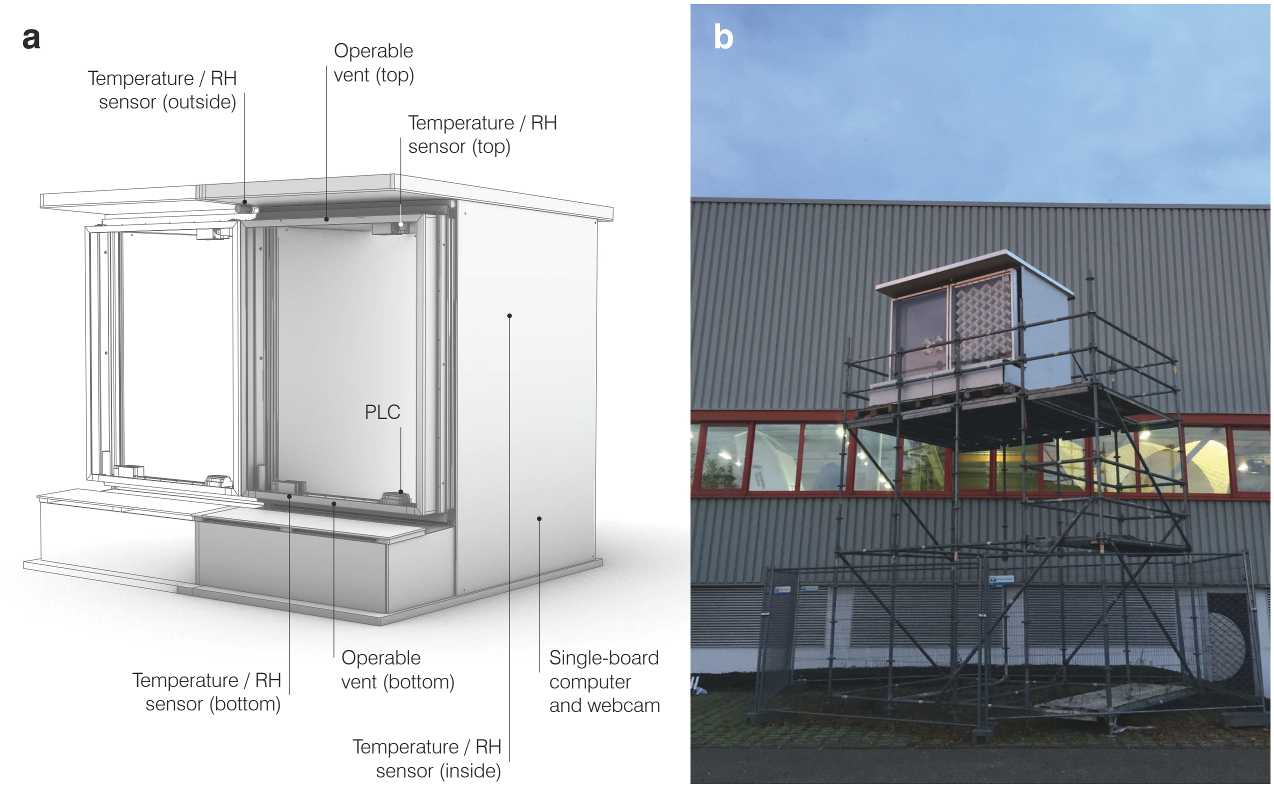

Supplementary Figure 2: Physical mock-up for long-term testing of the facade system. (**a**) The set-up consists of two modules of a window and enclosed room, each equipped with data collection infrastructure. (**b**) The facade mock-up emulates the height, orientation, and environmental conditions of the target building facade.

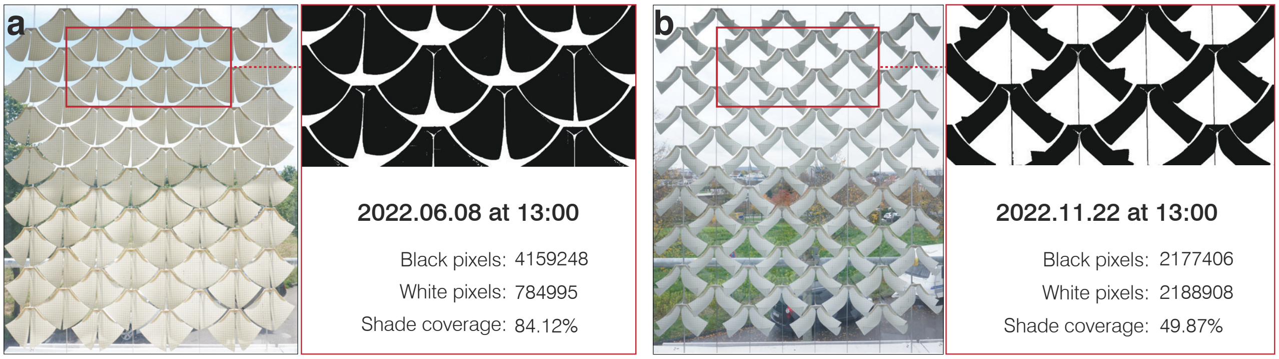

Supplementary Figure 3: Analysis of shade coverage during summer and winter. The original, photographed images of the 4D-printed modules are shown along with the result of black & white image processing. The data presented here are samples of (**a**) a hot summer day at 13:00 (**b**) and a cool winter day at the same time.

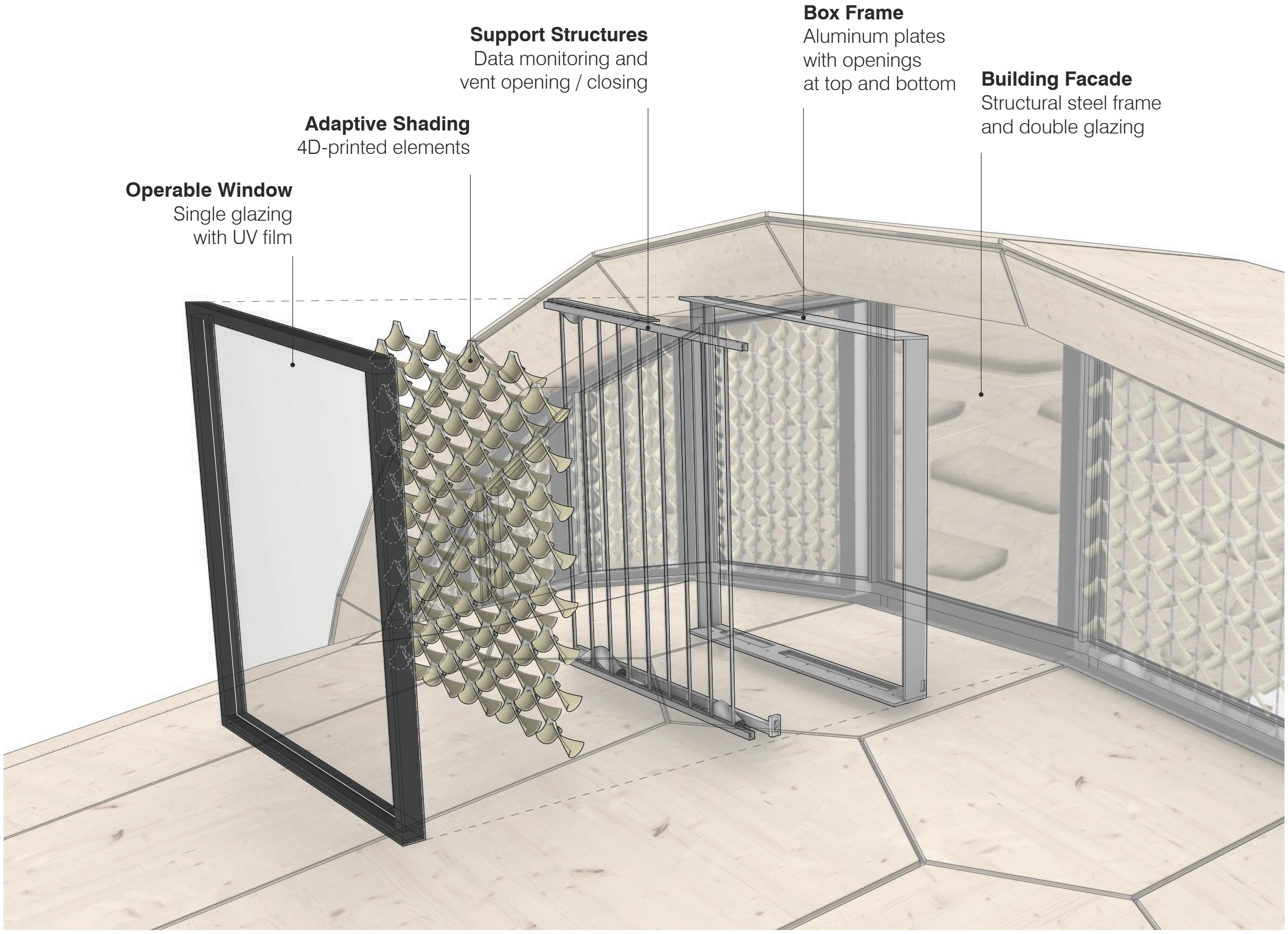

Supplementary Figure 4: The architectural integration of the self-shaping shading system is illustrated here with an exploded assembly of one window. These components are attached to the structural steel frame of the building facade.

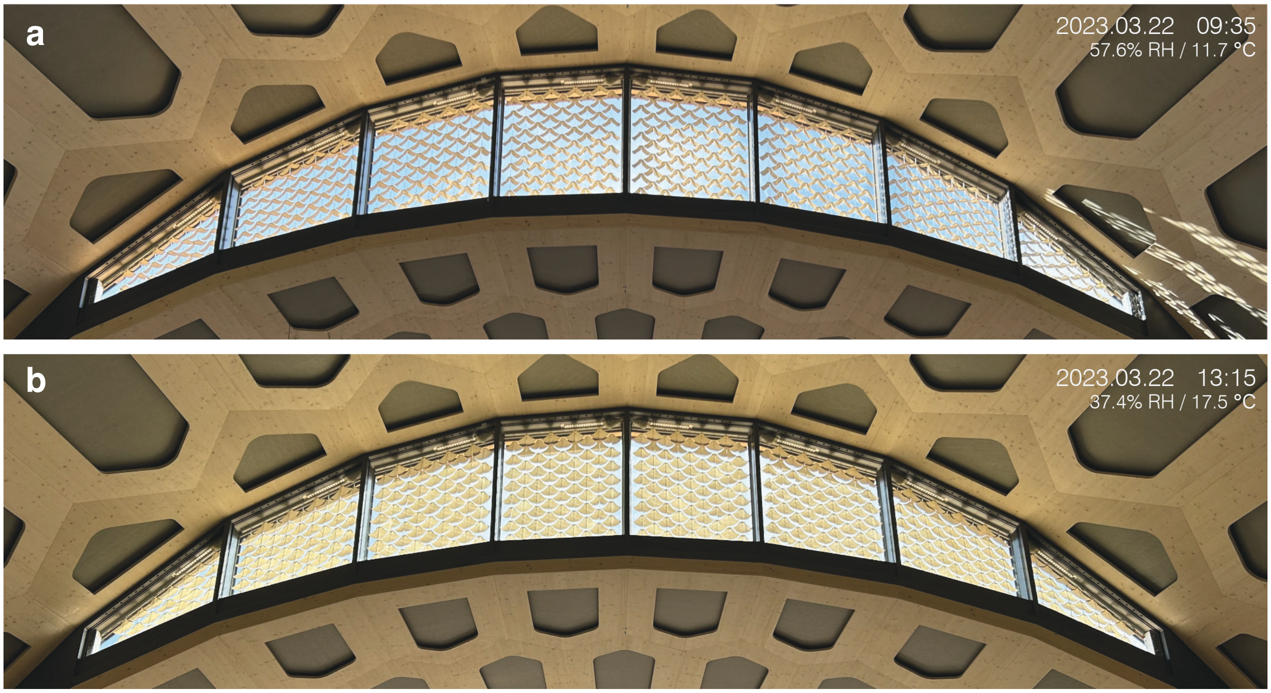

Supplementary Figure 5: Deployed on a real building facade, the 4D-printed shading system is shown on March 22nd (**a**) with an open configuration during the cool morning conditions of $11.7\,°\mathrm{C}$ and 57.6% RH, (**b**) and with a closed configuration during the warming afternoon conditions of $17.5\,°\mathrm{C}$ and 37.4% RH.
