# Supplementary material for: Weather-responsive adaptive shading through biobased and bioinspired hygromorphic 4D-printing
Source: Nat Commun. 2024 Nov 28;15:10366. doi: 10.1038/s41467-024-54808-8 (PMC11604995; doi:10.1038/s41467-024-54808-8)
Supplement: Supplementary file 2 — Description of Additional Supplementary Files [file 41467_2024_54808_MOESM2_ESM.pdf]

Description of Additional Supplementary Files

Supplementary Movie 1. Motion response of the 4D-printed self-shaping elements under real weather conditions in summer 2022, from May 31st to June 6th (time-lapse).

Supplementary Movie 2. Motion response of the 4D-printed self-shaping elements under real weather conditions in summer 2022, from July 21st to 27th (time-lapse).

Supplementary Movie 3. Motion response of the 4D-printed self-shaping elements under real weather conditions in winter 2022, from November 15th to 21st (time-lapse).

Supplementary Movie 4. Motion response of the 4D-printed self-shaping elements under real weather conditions in winter 2022, from December 8th to 14th (time-lapse).
